# Neuroinflammation regulates the balance between hippocampal neuron death and neurogenesis in an ex vivo model of thiamine deficiency

**DOI:** 10.1186/s12974-022-02624-6

**Published:** 2022-11-14

**Authors:** Larissa M. G. Cassiano, Marina S. Oliveira, Jeanne Pioline, Anna C. M. Salim, Roney S. Coimbra

**Affiliations:** 1 Neurogenômica, Imunopatologia, Instituto René Rachou, Fiocruz, Belo Horizonte, MG 30190-002 Brazil; 2 Pós-Graduação em Neurociências, Universidade Federal de Minas Gerais, Belo Horizonte, MG 31270-901 Brazil; 3 Aix-Marseille University, Marseille, France; 4 Plataforma de Sequenciamento NGS (Next Generation Sequencing), Instituto René Rachou, Fiocruz, Belo Horizonte, MG 30190-002 Brazil

**Keywords:** Thiamine deficiency, Organotypic hippocampal culture, Neurogenesis. inflammation, Neurodegeneration, Neuroregeneration

## Abstract

**Background:**

Thiamine (vitamin B1) is a cofactor for enzymes of central energy metabolism and its deficiency (TD) impairs oxidative phosphorylation, increases oxidative stress, and activates inflammatory processes that can lead to neurodegeneration. Wernicke–Korsakoff syndrome (WKS) is a consequence of chronic TD, which leads to extensive neuronal death, and is associated with neuropathological disorders, including cognitive deficits and amnesia. The hippocampus is one of the brain areas most affected by WKS. B1 replacement may not be enough to prevent the irreversible cognitive deficit associated with WKS.

**Materials and methods:**

An organotypic hippocampal slice culture (OHC) model was developed to investigate, using immunofluorescence and confocal microscopy and transcriptome analysis, the molecular mechanisms underlying the neurodegeneration associated with TD. The effect of anti-inflammatory pharmacological intervention with resveratrol (RSV) was also assessed in B1-deprived OHCs.

**Results:**

In OHCs cultured without B1, neuronal density decayed after 5 days and, on the 7th day, the epigenetic markings H3K4me3 and H3K9me3 were altered in mature neurons likely favoring gene transcription. Between the 7th and the 14th day, a pulse of neurogenesis was observed followed by a further massive neuron loss. Transcriptome analysis at day nine disclosed 89 differentially expressed genes in response to B1 deprivation. Genes involved in tryptophan metabolism and lysine degradation KEGG pathways, and those with Gene Ontology (GO) annotations related to the organization of the extracellular matrix, cell adhesion, and positive regulation of synaptic transmission were upregulated. Several genes of the TNF and FoxO signaling pathways and with GO terms related to inflammation were inhibited in response to B1 deprivation. *Nsd1*, whose product methylates histone H3 lysine 36, was upregulated and the epigenetic marking H3K36me3, associated with negative regulation of neurogenesis, was increased in neurons. Treating B1-deprived OHCs with RSV promoted an earlier neurogenesis pulse.

**Conclusion:**

Neuroregeneration occurs in B1-deficient hippocampal tissue during a time window. This phenomenon depends on reducing neuroinflammation and, likely, on metabolic changes, allowing acetyl-CoA synthesis from amino acids to ensure energy supply via oxidative phosphorylation. Thus, neuroinflammation is implicated as a major regulator of hippocampal neurogenesis in TD opening a new search space for treating WKS.

**Supplementary Information:**

The online version contains supplementary material available at 10.1186/s12974-022-02624-6.

## Introduction

Thiamine, or vitamin B1, is an essential vitamin, therefore should be acquired through diet [1]. B1 is implicated in several processes including the biosynthesis of lipids, nucleic acids, branched-chain amino acids and some neurotransmitters, such as γ-aminobutyric acid (GABA) and glutamate [2]. Its active form, thiamine pyrophosphate (TPP), is a cofactor of important enzymes involved in energy metabolism (Additional file 1), such as transketolase (TK), pyruvate dehydrogenase complex (PDHC), branched-chain α-keto acid dehydrogenase complex (BCKDC) and α-ketoglutarate dehydrogenase complex (KGDHC) [3]. As a result, thiamine deficiency (TD) compromises oxidative phosphorylation, increases oxidative stress and activates inflammatory processes that can lead to cell death [4].

Wernicke–Korsakoff syndrome (WKS) manifests as a consequence of the non-ingestion or malabsorption of vitamin B1 and is characterized by cognitive deficits, amnesia, neuropathological disorders with extensive neurodegeneration, and cellular and molecular dysfunction. In patients with WKS, damage is observed in several areas of the central nervous system (CNS) such as the hippocampus, thalamus, mammillary bodies, and cerebellum (reviewed in [5–7]). WKS has been related to two clinical stages: Wernicke’s encephalopathy (WE) due to acute vitamin B1 deficiency and characterized by ataxia, ophthalmoplegia and confusion; and Korsakoff psychosis, a chronic stage of TD characterized by frontal lobe dysfunction, affective disorders, and anterograde/retrograde amnesia [2, 8].

The etiology of WKS is mainly related to alcoholism [9] and the prevalence of classical symptoms associated with WKS in chronic alcoholic patients is more than 10% [10, 11]. However, the syndrome is underdiagnosed in about 75 to 80% of cases, which are identified only after the patient's autopsy [10, 12]. The lesions caused by TD can lead to patient’s death, with reported mortality rates of 17–20%, or, in 85% of the cases, to the development of Korsakoff psychosis (reviewed in [13]). Early and urgent intervention with appropriate B1 treatment can facilitate the recovery, however the cognitive deficits in survivors, especially in alcoholic patients, may remain [14].

The hippocampus is a brain structure that plays an important role in the creation of new declarative memories and in the development of spatial memories, contributing to the formation of a cognitive map of the environment and context-dependent memory [15, 16]. It is one of the brain areas most affected by TD and WKS [17]. This structure also harbors one of the areas responsible for CNS neurogenesis in post-embryonic life: the granular layer of the dentate gyrus, where resident self-renewable neural progenitor cells (NPCs) can differentiate into neurons, oligodendrocytes and astrocytes (reviewed in [18]).

Several studies have investigated TD in rats using vitamin B1-deficient diet and daily intraperitoneal injections of pyrithiamine, a substance that inhibits thiamine absorption [19]. Aiming at reducing animal use and suffering, an organotypic hippocampal slice culture model (OHC) was developed, which preserves diversity and interconnections between cells and their physiological viability, to investigate the molecular mechanisms underlying the imbalance between neuronal death and neurogenesis associated with TD in the brain.

## Materials and methods

### Animals

OHCs were prepared from infant (7 days old) Wistar rats, obtained from the Institute of Science and Technology in Biomodels (ICTB) (FIOCRUZ). Animals were housed ten per cage with a lactating female in controlled temperature and humidity (20–26 °C, 40–60%, respectively), under a 12:12 h light–dark cycle and given water and food ad libitum. All the experimental procedures were approved by the FIOCRUZ Animal Ethics Committee (license LW-10/18).

### Organotypic hippocampal slice cultures (OHC)

OHCs were prepared using the method described by Stoppini and collaborators [20] with modifications. Briefly, after euthanasia by decapitation, brains were rapidly removed and both hippocampi were dissected in ice-cold basic medium with 75% MEM Eagle + Hepes (Vitrocell Embriolife, Campinas, Brazil) and 25% Hank’s balanced salt solution 1× (Sigma Aldrich, Saint Louis, MO). Hippocampi were transversally cut in 400-μm-thick sections using a McIlwain tissue chopper (Mickle Laboratory Engineering Co Ltd., Gomshal, United Kingdom) and the slices were transferred (six slices from different animals per well) onto Millicell cell culture inserts (PICM0RG50) (Merck, DarmstaTD, Germany) in 6-well culture plate.

OHCs were incubated at 37 °C, 5% CO_2_ in 1 mL of nutrition medium constituted by 50% MEM Eagle + Hepes, 25% Hank’s balanced salt solution 1× and 25% heat-inactivated horse serum (Bio Nutrientes, Barueri, Brazil). After 7 days, the medium was replaced with a serum-free basic medium, since serum affects in vitro neurogenesis [21], and cultures were further incubated until the 14th day to complete stabilization.

After the stabilization phase, the OHCs were arranged in TD and control groups, and the TD cultures were further cultured in B1-free basic medium until the end of each experiment.

During the stabilization and challenging phases, 50% of the medium volume was replaced with fresh medium every 2 or 3 days to renew the necessary nutrients.

#### Assessing neurogenesis in the OHCs by 5-bromo-2'-desoxyuridine incorporation

After 7 days, OHCs from TD and control groups were treated with 10 µM 5-bromo-2'-desoxyuridine (BrdU) (Sigma Aldrich) in basic medium for 48 h. After that, OHCs were washed with sterile PBS 1× for 3 times and further cultured in basic medium until day 10.

### Immunofluorescence

The OHCs were immune-stained according to Gogolla and collaborators [22] using 0.05% Tween in PBS as permeabilization solution. Mature neurons were labeled with rabbit or mouse antibodies conjugated to the Cy3 fluorophore (Merck, #ABN78C3 or #MAB377C3, 1:100). Activated microglia and trimethylation of different lysine residues of histone H3 were indirectly labeled with primary antibodies anti-Iba1 (Abcam, #AB178847, 1:100), anti-H3K4me3 (Merck, #05-1339, 1:500), anti-H3K9me3 (Merck, #07-442, 1:500) and anti-H3K36me3 (Abcam, #AB9050, 1:200), respectively. As secondary antibodies, anti-rabbit (Thermo Fisher, #A11034, 1:400) or mouse (Thermo Fisher #A10684, 1:20,000) IgG conjugated with Alexa Fluor 488 fluorophore were used.

DNA fragmentation in apoptotic cells was assessed with the TUNEL assay (Thermo Fisher), performed according to the manufacturer’s instructions.

For BrdU detection, cultures were incubated in HCl 1 M for 30 min after permeabilization and stained with mouse anti-BrdU antibody conjugated to Alexa Fluor 488 (Merck, #FCMAB101A4, 1:100).

The OHCs were counterstained with DAPI (Thermo Fisher) and mounted onto glass microscope slides with ProLong Diamond Antifade Mountant (Thermo Fisher).

### Confocal microscopy

Z-series image stacks (~ 12 optical images 2.79 µm each) of 10× amplification of the immunostained OHCs were obtained using a Nikon Eclipse Ti confocal microscope (Nikon, Tokyo, Japan) with wavelength filter 488/561. The threshold parameters of each laser were adjusted with a negative control (without primary antibody) to remove background noise and tissue autofluorescence. For acquisition, OHCs regions with greater neuronal (corresponding to NeuN+ fluorescence) or activated microglia (corresponding to Iba1+ fluorescence) densities were chosen. The z-series image stacks were deconvoluted and analyzed using NIS-Elements Analysis software tools. Briefly, the Cy3 channel (corresponding to NeuN+ fluorescence) was treated with the *Noise Reduction*, *Gauss–Laplace Sharpen* and *Local Contrast* tools. The total tissue area was determined by an automatic binary mask under the DAPI channel, allowing the delimitation only of spaces filled with tissue. For neuron counting, another binary mask was created, based on the previous field, where only elements of the Cy3 channel with > 10 A.U fluorescence threshold were selected and quantified, yielding the neuronal density value (NeuN+/mm^2^). The same was done for the activated microglia count, where only areas with signal of the Alexa Fluor 488 channel with a fluorescence threshold > 10 A.U. were selected and measured, yielding the activated microglia density value (Iba1+/mm^2^). Skeleton Analysis method developed by Young et al*.* [23] was used to access Iba1+ microglial cells ramification and branch lengths. The data were represented as endpoints (cell branch tips) normalized by branch lengths, where the highest values represent the less activated microglial state (cells with more branched morphology and smaller size). Finally, neurons with double tagging (NeuN+ and TUNEL+, BrdU+, H3K4me3+, H3K9me3+ or H3K36me3+) were quantified using the *Interest Region Manager (ROI)* tool, where regions of intersection between the Cy3 and Alexa Fluor 488 channel tags were detected allowing the calculation of the percentage of mature TUNEL+ neurons (%NeuN+ TUNEL+), post-mitotic neuron density (BrdU+ neurons/mm^2^) and the fluorescence intensity (IF) for H3 trimethylation at different lysine residues normalized by the number of positive neurons for these epigenetic markings (Sum IF H3K_me3/NeuN+H3K_me3+).

### Transcriptomic profiling

RNA was obtained from pools of three OHCs, from three different animals, cultured with or without B1 for 9 days. Total RNA was extracted using miRNeasy Mini Kit (Qiagen, Hilden, Germany), according to the manufacturer’s instructions. All samples had their RNA quantified by fluorometry using Qubit RNA HS Assay Kit (Invitrogen, Carlsbad, CA) with Qubit 2.0 Fluorometer (Invitrogen, Carlsbad, CA) and had their quality assessed by capillary electrophoresis with a Bioanalyzer 2100 (Agilent, Santa Clara, CA).

Later, libraries were produced using the TruSeq Stranded mRNA Kit (Illumina, San Diego, CA) and the indexed fragments were sequenced using the TG NextSeq 500/550 High Output Kit v2 (Illumina, San Diego, CA).

### Bioinformatic analyses

Raw reads were processed with Trimmomatic [24] to remove adaptors and low quality bases or too short reads (less than 36 nt). Processed reads were aligned to *Rattus norvegicus* (release 94) reference genome using STAR [25] and uniquely localized reads were used to calculate reads per kilobase of transcript, per million mapped reads (RPKM) values [26] for each gene. Contrast analysis between TD and control groups was performed using DESeq2 software [27]. Fold change was calculated for each sample based on the mean of the three samples in control group. Genes with fold change greater than 1.5 and adjusted *P* values lower than 0.01 were considered to be differentially expressed.

The samples and the differentially expressed genes were hierarchically clustered with Spearman correlation as a comparison method and average linkage using GenePattern [28]. Functional Enrichment analysis of differentially expressed genes was performed with the web-based software Database for Annotation, Visualization and Integrated Discovery (DAVID) v6.8 at the National Institute of Allergy and Infectious Diseases (NIAID), NIH (https://david.ncifcrf.gov/tools.jsp) [29] and the Ingenuity Pathways Analysis (IPA) (Qiagen).

### Real‐time qPCR

RNA samples were obtained, as described above, from pools of six OHCs (two per animal) cultured with or without B1 for 4, 7 or 9 days. The cDNA was synthesized from 0.5 to 1 μg total RNA using High-Capacity cDNA Reverse Transcription Kit (Applied Biosystems), according to the manufacturer’s protocol. Rat-specific TaqMan Gene Expression Assays (Applied Biosystems) were used to detect *Nsd1* (Nuclear Receptor Binding SET Domain Protein 1) (Rn01441947_m1), *Bmp4* (Bone Morphogenetic Protein 4) (Rn00432087_m1), *Hes5* (Hes Family BHLH Transcription Factor 5) (Rn00821207_g), *Ogdh* (oxoglutarate dehydrogenase) (Rn01443655_m1), *Mmp9* (Matrix Metalloproteinase 9) (Rn00579162_m1), *Dcx* (Doublecortin) (Rn00570390_m1), *Neurod1* (Neuronal Differentiation 1) (Rn01280117_m1), *C3* (Complement C3) (Rn00566466_m1) and *Lcn2* (Lipocalin 2) (Rn00590612_m1). For all RT-qPCR assays the expression levels of target genes were normalized to the levels of the housekeeping gene *Ppia* (peptidylprolyl isomerase A) (Rn00690933_m1). Each well was loaded with cDNA (4 ng/µL) mixed with TaqMan Fast Advanced Master Mix (Thermo Fisher) in a total volume of 10 μL. Thermal cycling and florescence detection was performed using the Applied Biosystems ViiA 7 Real-Time PCR System (ThermoFisher) according to the manufacturer’s protocol**.** The relative gene expression was calculated using the 2^−ΔΔCt^ method [30].

### Pharmacological intervention with an anti-inflammatory drug

Pharmacological intervention with anti-inflammatory resveratrol (RSV) was used to test the causal relationship between neuroinflammation and neurogenesis in the OHCs model of TD. The OHCs were incubated with resveratrol (RSV) (Sigma Aldrich) at 10, 50 or 100 µM for 3 days starting at day 4 of incubation with or without B1.

### Statistical analyses

The statistical analyses were performed using GraphPad Prism (version 6.01) (GraphPad Software Inc., Irvine, CA). Statistical tests were chosen according to the experimental design and data distribution: (1) Kruskal–Wallis test followed by the multiple comparison test of Dunn was used to compare three or more groups with non-parametric distribution; (2) two-tailed Mann–Whitney test was used to compare two groups with non-parametric distribution; (3) two-tailed *T* test was used to compare two groups with parametric distribution; (4) two-way ANOVA followed by Tukey’s test was used when comparisons involved the effect of two factors on a dependent variable in three or more groups. Data were expressed as median ± interquartile range or mean ± standard deviation. Differences were considered significant when *P* values were smaller than 0.05.

## Results

### Phenotypic features associated with TD in OHCs

**Fig. 1:**
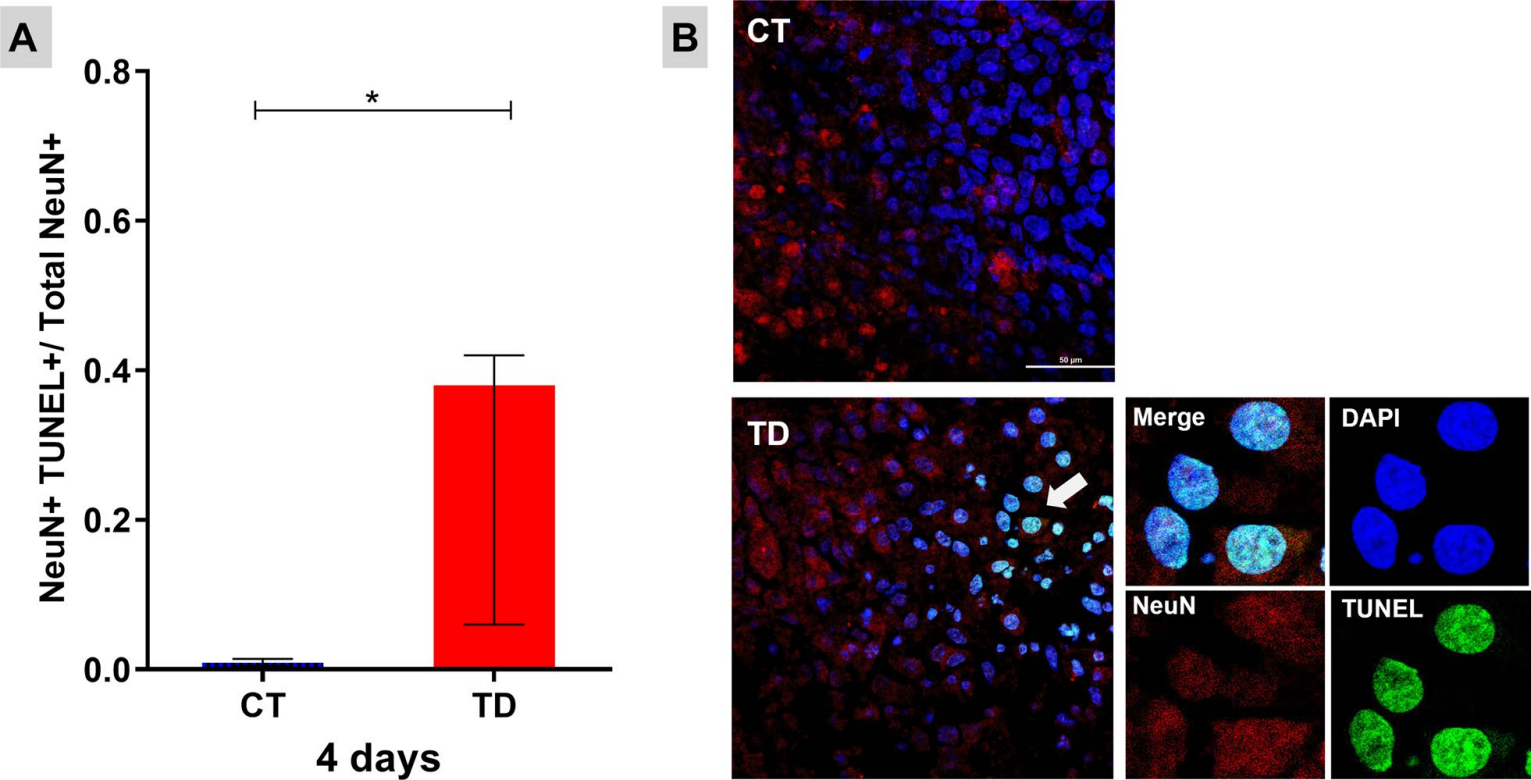
Mature neurons die by apoptosis in TD. TUNEL assay detect DNA fragmentation during apoptosis. **A** Fraction of TUNEL-labeled neurons after 4 days of B1 deprivation. Data were expressed as median ± interquartile range. Mann–Whitney test was used (*P ≤ 0.05). **B** Confocal microscopy images (×60) of OHCs show mature neurons (NeuN+) (in red), cell nuclei (DAPI) (in blue), and TUNEL (in green). In detail, a digital zoom (×5.5) of typical apoptotic neurons. *CT* controls, *TD* thiamine deficiency (scale bar = 50 μm)

**Fig. 2:**
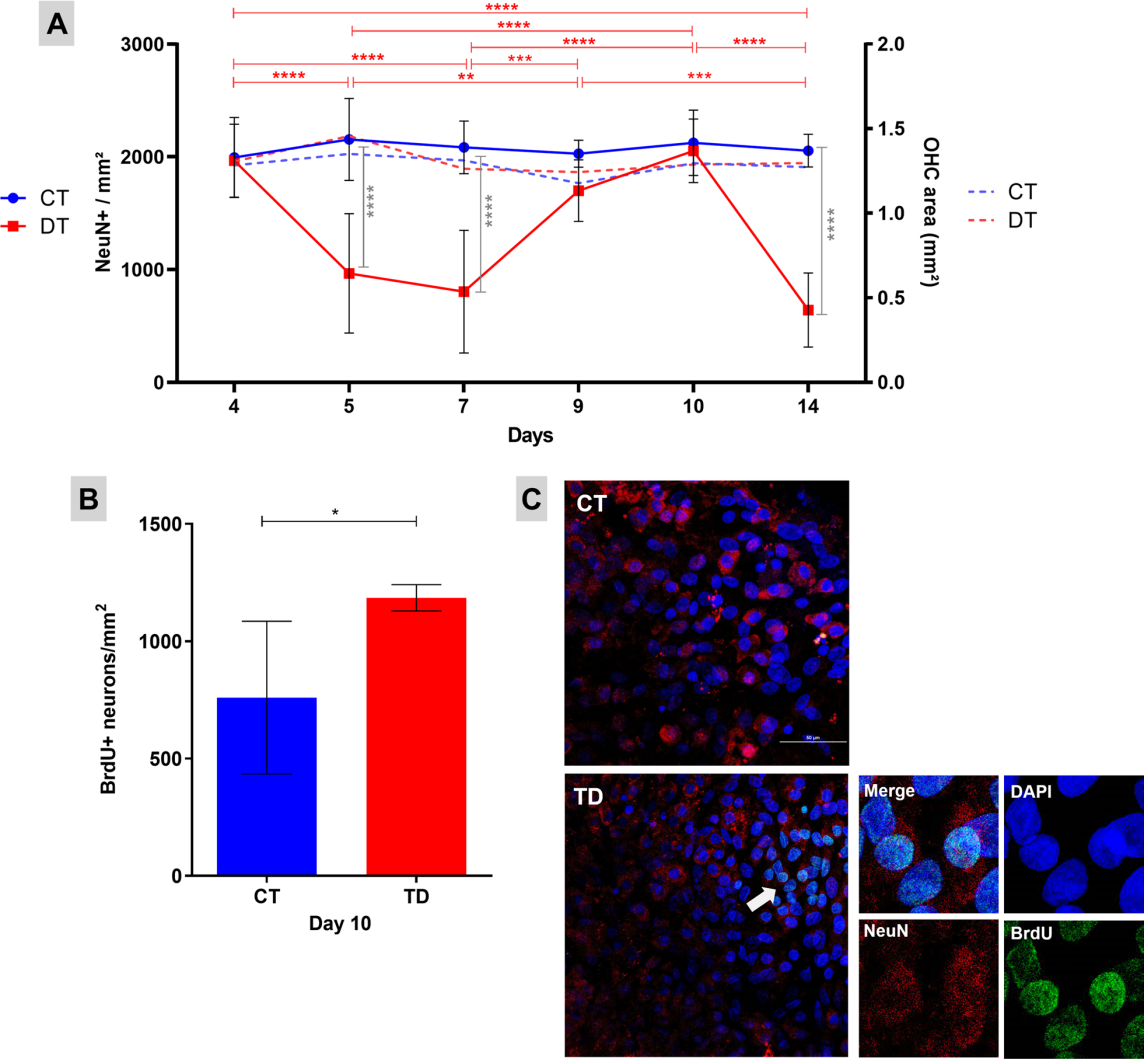
Neuronal density in OHCs at different timepoints of B1 deprivation. OHCs challenged with B1 deprivation lost approximately 71% of neurons from the 5th day on. Surprisingly, between the 7th and 14th day of culture without B1, a pulse of neurogenesis followed by a new massive loss of neurons was observed. **A** Left *Y* axis shows the neuronal density means (NeuN+/mm^2^) of the CT (controls) (*n* = 12) and TD (thiamine deficiency) (*n* = 12). Groups were compared with the bidirectional analysis of variance (ANOVA) test followed by the Tukey’s multiple comparisons test. Data were expressed as mean ± standard deviation (*P ≤ 0.05, **P ≤ 0.01, ***P ≤ 0.001, ****P ≤ 0.0001). Statistical differences represented by the red horizontal bars and asterisks refer to the variation in the TD group between the different culture time points. Gray vertical bars and asterisks refer to the intergroup differences (CT versus TD) at the same timepoint**.** Right Y axis shows the OHC area means of the CT (*n* = 12) and TD (*n* = 12). Groups were compared with the bidirectional ANOVA test followed by the Tukey's multiple comparisons test. **B** BrdU assay identify proliferating cells. The graph shows post-mitotic neurons density (BrdU+ neurons/mm^2^) between days 7 and 10 of B1 deprivation. N sample: CT (*n* = 3) and TD (*n* = 4). Two-tailed *T* test was used (*P ≤ 0.05) and data were expressed as mean ± standard deviation. **C** Confocal microscopy images (×60) of OHCs show mature neurons (NeuN+) (in red), cell nuclei (DAPI) (in blue), and BrdU (in green). In detail, a digital zoom (×8) of typical BrdU staining. *CT* controls, *TD* thiamine deficiency (scale bar = 50 μm)

After 4 days of B1 deprivation, an increase in the number of TUNEL+ and NeuN+ neurons were observed with immunofluorescence and confocal microscopy analyses (Figs. 1A and 2A). At the 5th day, 71% reduction in neuronal density was observed in OHCs cultured without B1 when compared to the control group (Fig. 2A). Surprisingly, a pulse of neurogenesis occurred later on since at the 10th day of B1 deprivation (Fig. 2B) and the density of mature neurons returned to the control levels (Fig. 2A). However, this phenomenon is followed by a new massive decrease of neuron population (Fig. 2A).

The OHCs repopulation with new neurons at day 10 of thiamine deprivation was confirmed by the BrdU incorporation assay. Figure 2B shows an increase in post-mitotic neurons density (BrdU+ neurons/mm^2^) between 7 and 10 days of B1 deprivation, suggesting that NPCs could survive and differentiate into new neurons during TD.

These results prove the existence of a temporal window when neuroregeneration is possible in the B1-deficient hippocampal tissue. The average area (mm^2^) of the OHCs did not change significantly along the culture period, indicating that the observed changes in the neuronal density in response to B1 deprivation are not due to OHC compaction (Fig. 2A).

**Fig. 3:**
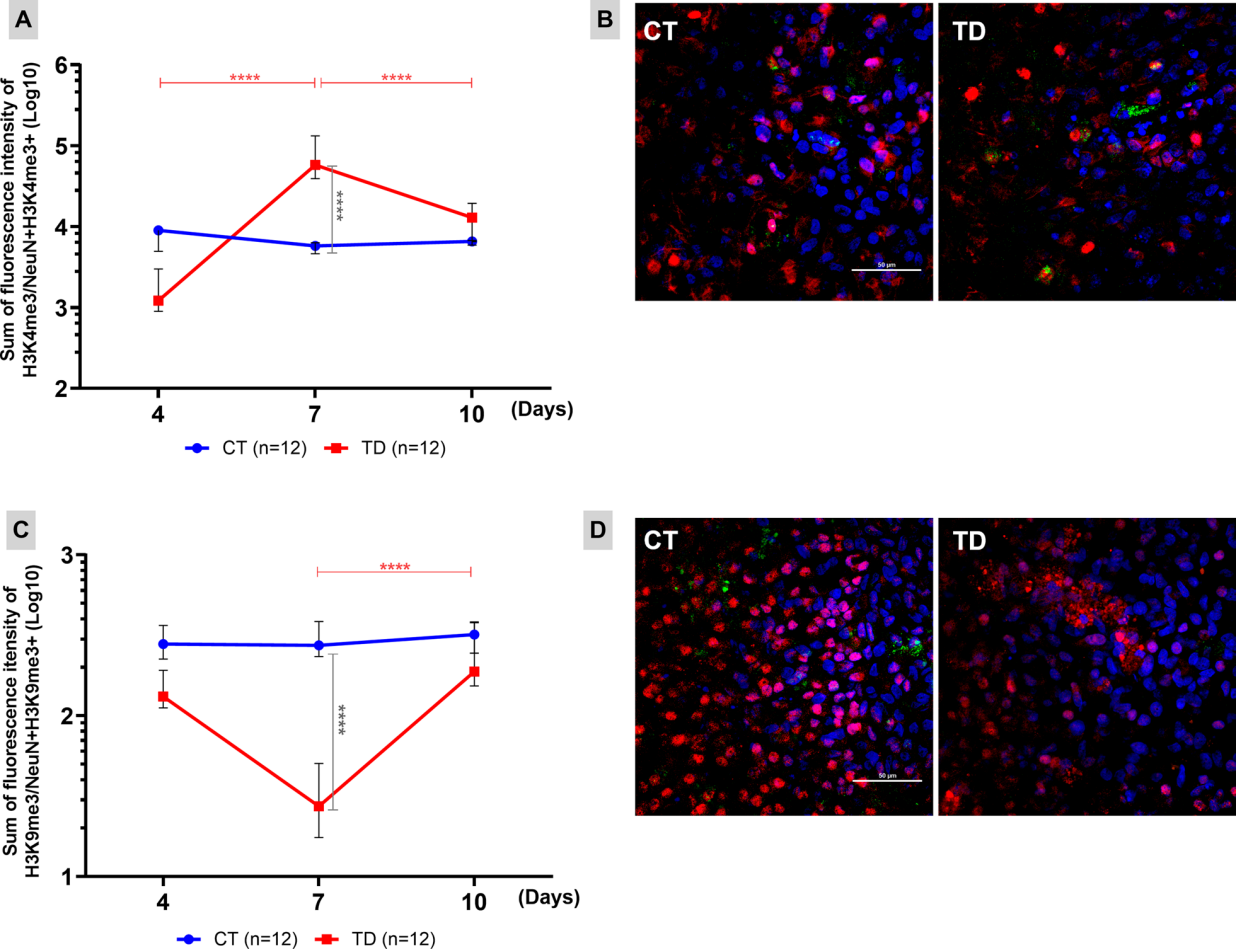
Epigenetic markings favor chromatin accessibility and gene expression during TD. The trimethylation of histone H3 lysines 4 and 9 modulate chromatin accessibility increasing or inhibiting gene expression, respectively. **A**, **C** Sum of the fluorescence intensity of the brand H3K4me3 (**A**) and H3K9me3 (**B**) normalized by the number of mature neurons identified with these markings, after 4, 7 and 10 days of culture with or without B1. The means of the CT (controls) and TD (thiamine deficiency) groups were compared with the bidirectional analysis of variance (ANOVA) test followed by the Tukey’s multiple comparisons test. Data were expressed as ± standard deviation (****P ≤ 0.0001). The statistical differences represented by red horizontal bars and asterisks refer to the variation in the TD group between the different timepoints. Gray vertical bars and asterisks refer to the intergroup differences (CT versus TD) at the same timepoint. **B**, **D** Confocal microscopy images (×60) of COH with markings of mature neurons (NeuN+) in red, cell nucleus (DAPI) in blue and epigenetic markings H3K4me3 (**B**) or H3K9me3 (**D**) in green after 7 days of culture with or without B1 (scale bar = 50 μm)

The epigenetic regulation of gene expression also seems to play an important role in the pathophysiology of TD. Figure 3 shows the fluorescence intensity of H3K4me3 (Panel A) and H3K9me3 (Panel B) normalized by the number of mature neurons (NeuN+) with these markings after four, seven and 10 days of culture with or without B1. The increase in the H3K4me3 marking and the decrease in H3K9me3 on the 7th day of B1 deprivation indicate an epigenetic regulation of chromatin status favorable to increased gene expression.

### Transcriptomic profiling

**Fig. 4:**
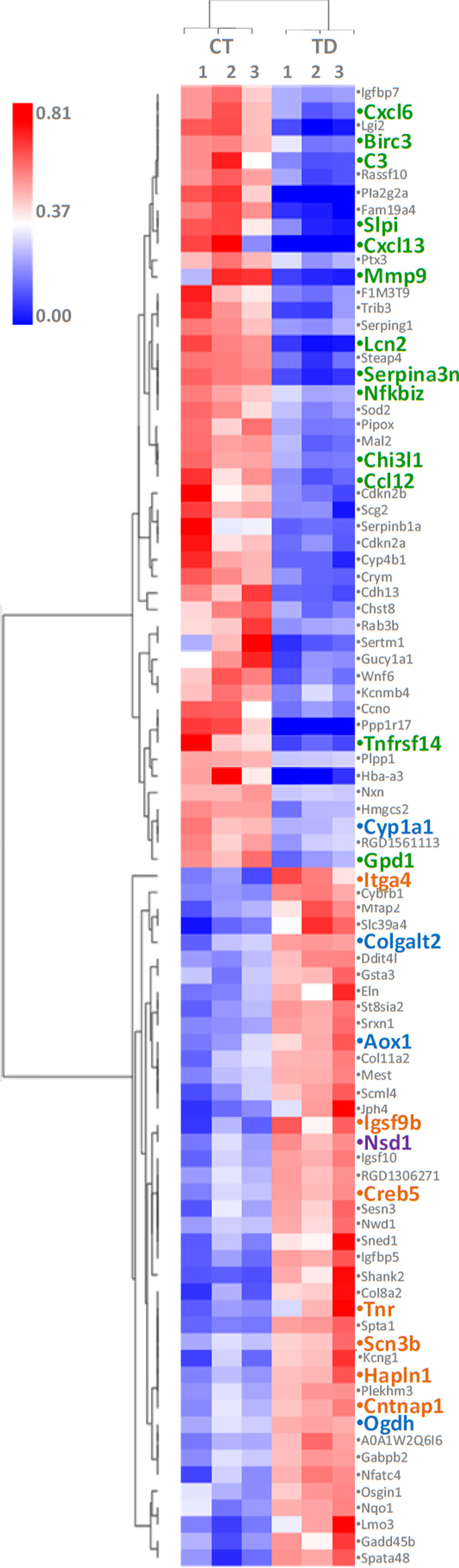
Hierarchical clustering and expression levels of the 89 differentially expressed genes in the TD group compared to the control. Fold change values were compared by Spearman correlation, average linkage and heatmap colors were normalized by the global *Z*-score. Among the genes differentially expressed in the contrast between TD and CT, genes related to inflammatory response (green), cell metabolism (blue), cell cycle, cell differentiation and survival (orange) and epigenetic regulation of neurogenesis (purple) were observed. *CT* controls, *TD* thiamine deficiency

Despite decades of research on TD, this is the first study to report a hippocampal neuroregeneration time window during B1 deprivation. To elucidate the mechanisms underlying this phenomenon, the transcriptional profiles of OHCs cultured for 9 days without and with B1 were compared. The contrast analysis disclosed 89 differentially expressed genes, 42 upregulated and 47 downregulated in TD group compared to the controls (Fig. 4 and Additional file 2). Functional enrichment analysis with DAVID software (Additional file 3) revealed that the set of genes with increased expression in TD is enriched in Kyoto Encyclopedia of Genes and Genomes (KEGG) pathways of Tryptophan metabolism (map00380), Lysine degradation (map00310), ECM (extracellular matrix)–receptor interaction (map04512), PI3K–Akt (phosphatidylinositol 3-kinase–protein kinase B) signaling pathway (map04151) and focal adhesion (map04510). This subset of genes was also found to be enriched in GO *(Gene Ontology)* annotations related to positive regulation of synaptic transmission (GO:0050806), nervous system development (GO:0007399), regulation of synaptic plasticity (GO:0048167), and long-term synaptic potentiation (GO:0060291). Importantly, the *Nsd1* gene, upregulated in TD, is annotated with GO terms related to the inhibition of neurogenesis by regulating the methylation of the lysine 36 at the histone H3 (positive regulation of transcription, DNA-templated [GO:0045893]; regulation of histone H3-K36 methylation [GO:0000414]; regulation of peptidyl-serine phosphorylation [GO:0033135]; regulation of RNA polymerase II regulatory region sequence-specific DNA binding [GO:1903025]).

At the same time, among the genes downregulated by TD, several molecules of the KEGG pathways of Glycerophospholipid metabolism (map00564), TNF (tumor necrosis factor) signaling pathway (map04668), Cell cycle (map04110), and FoxO (Forkhead box class O) signaling pathway (map04068) and have GO annotations related to inflammatory response (GO:0006954), immune response (GO:0006955), chemokine-mediated signaling pathway (GO:0070098), cellular response to TNF (GO:0071356), and cellular response to IL-1 (GO:0071347)*.*

Functional enrichment analyses performed with IPA software (Additional file 4) indicated inhibition of canonical pathways related to neuroinflammation signaling. NFE2L2 (nuclear factor erythroid 2-related factor 2), CREB1 (cAMP responsive element binding protein 1), IFNG (interferon gamma), TNF and TGFB1 (transforming growth factor beta 1) were predicted as potentially inhibited molecules in the OHCs of the TD group. Moreover, the results obtained with IPA indicated the decrease in the expression of APP as a possible central regulator for the reduction of neuroinflammation in OHCs challenged with B1 deprivation.

### Time series analysis of differentially expressed genes at day 9 of B1 deprivation selected from transcriptome analysis and related to inflammation, energy metabolism and neurogenesis

**Fig. 5:**
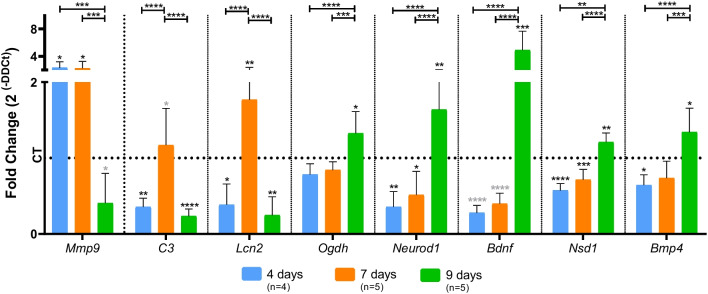
Relative expression of selected genes in OHCs cultured with or without B1. Fold change values were calculated from RT-qPCR data as 2^(−ΔΔCt)^, therefore, the dotted line indicates the basal mean expression level of the respective gene in the control group (CT). The means of the CT and thiamine deficiency (TD) groups were compared with the bidirectional analysis of variance (ANOVA) test followed by Tukey’s multiple comparisons test. CT and TD groups were also analyzed separately with Student’s *t*-test (*P* value in light gray) to identify possible statistical differences not detected by the multiple comparison method due to low statistical power of the small sample. Data were expressed as mean ± standard deviation (*P ≤ 0.05, **P ≤ 0.01, ***P ≤ 0.001, ****P ≤ 0.0001)

Transcriptome analysis revealed some key genes potentially related to the neuroregeneration phenotype observed in OHCs cultured for 9 days without B1. Then, the expression levels of some of these genes were assessed by RT-qPCR after 4, 7 and 9 days of culture with or without B1 (Fig. 5). The inflammatory genes, *C3*, *Lcn2*, and *Mmp9* were upregulated in response to B1 deprivation, when massive neuron loss was observed in the OHCs, and downregulated at day 9 during the neurogenesis time window; an inverse expression pattern was observed for *Bdnf*, *Bmp4*, *Neurod1*, *Nsd1* and *Ogdh* at these same time points*.* It is noteworthy that, at day 9 of B1 deprivation, *Mmp9* expression strongly and negatively correlates with the expression of *Bmp4*, *Bdnf*, *Neurod1*, and *Ogdh.* At this same time point, positive correlation was also observed between the expression levels of *Nsd1* and *Bmp4*, and *Bdnf* and *Ogdh* (Additional file 5)*.* Altogether, these results validate the transcriptomic and phenotypic findings and add an additional confidence level to the hypothesis that reduced inflammation is required for the neurogenesis pulse observed in this experimental model.

### Assessment of the H3K36me3 epigenetic marking related to neurogenesis suppression

**Fig. 6:**
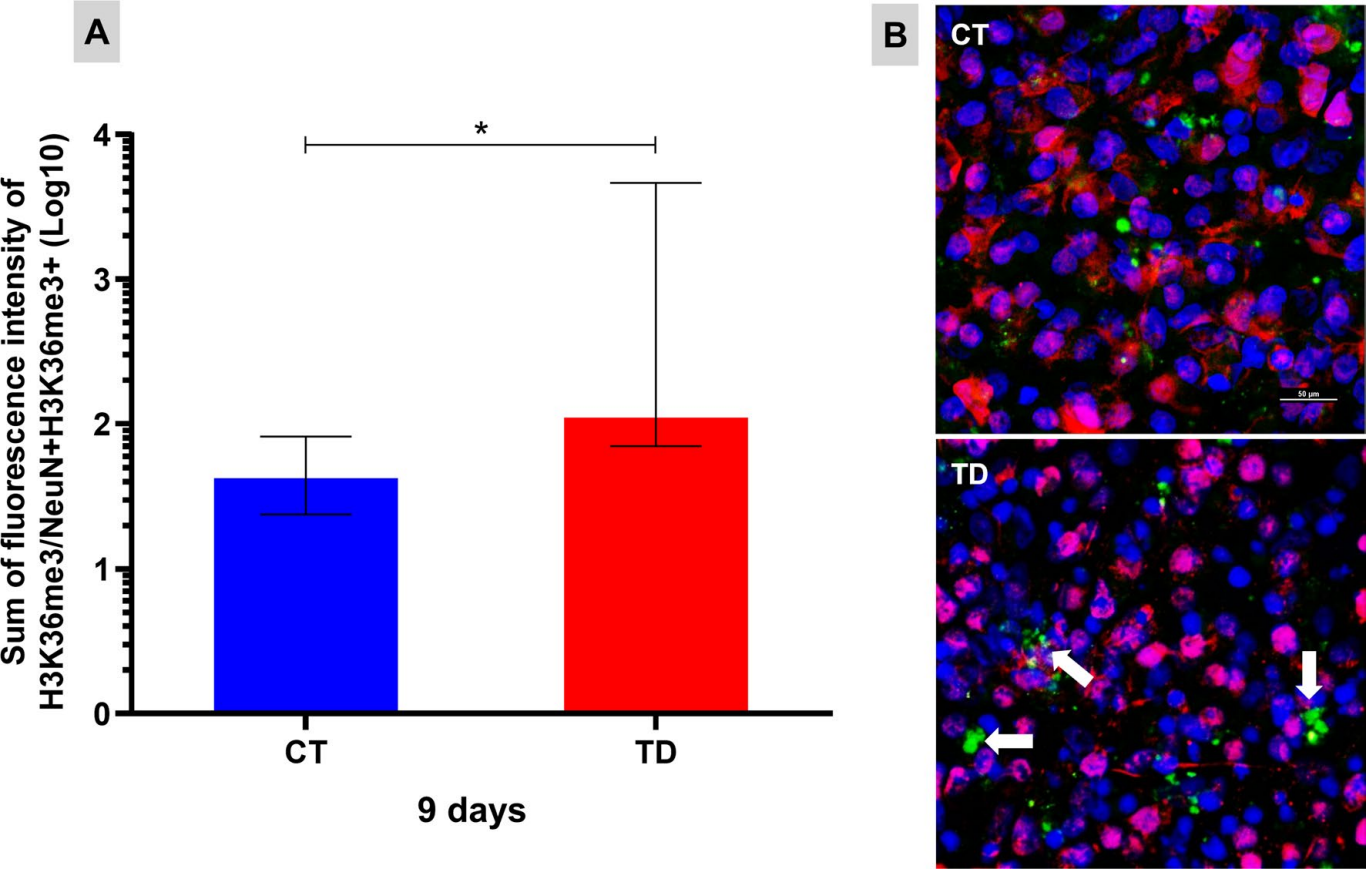
Epigenetic marking that inhibits neurogenesis after OHCs is repopulated with new neurons in advanced TD. The trimethylation of histone H3 lysine 36 (arrows in **B**) positively regulates the expression of the *Bmp4* gene, an important inhibitor of neurogenesis. **A** Sum of the fluorescence intensity of H3K36me3 normalized by the number of mature neurons identified with this marking after 9 days of culture with or without B1. The medians of the CT (controls) and TD (thiamine deficiency) groups were compared with the Mann–Whitney test and data were expressed as median ± interquartile. (*P ≤ 0.05). **B** Confocal microscopy images (×100) of OHCs labeled for mature neurons (NeuN+) in red, cell nucleus (DAPI) in blue and H3K36me3 in green after 9 days of culture with or without B1 (scale bar = 50 μm)

Functional enrichment analyses also indicated a process of inhibition of neurogenesis by epigenetic regulation after repopulation observed in OHCs cultured at 9 days in B1 deprivation. The protein encoded by the *Nsd1* gene is responsible for the mono and demethylation of lysine 36 at the histone H3. The trimethylation of this residue in H3, in turn, may increase the expression of the *Bmp4* gene, an important inhibitor of neurogenesis. Figure 6A shows the fluorescence intensity for H3K36me3, normalized by the number of mature neurons (NeuN+) with this epigenetic marking, after 9 days of culture with or without B1. The increased fluorescence intensity of H3K36me3 suggests that, once OHCs are repopulated with new neurons, differentiation is switched off in these cells.

### Anti-inflammatory pharmacological intervention

In order to shed light on the role of the inflammatory response to TD in the regulation of the balance between neuron death and neurogenesis in the hippocampus, the OHCs were treated with three different doses of the RSV (5, 50 or 100 μM) starting at the 4th day of culture with or without B1. The effects of RSV on microglia activation and neuron density were accessed and quantified by immunofluorescence and confocal microscopy after 3 days of treatment.

**Fig. 7:**
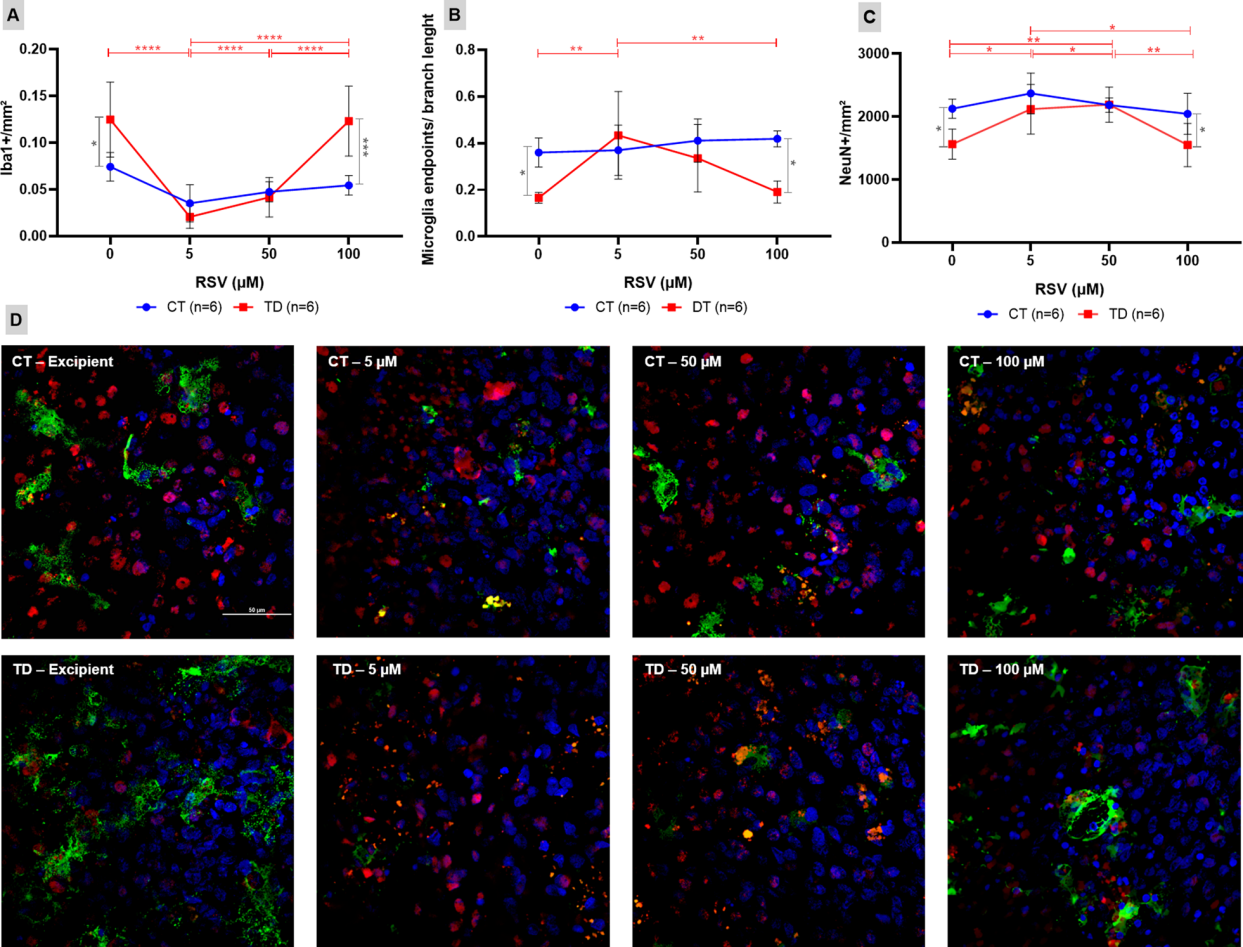
RSV prevents microglia activation and promotes an earlier neurogenesis pulse in B1-deprived OHCs. **A**–**C** The means of activated microglia (**A**), microglia endpoints/branch length ratio (**B**), and neuronal density (**C**) of the CT (controls) and TD (thiamine deficiency) groups were compared with the bidirectional analysis of variance (ANOVA) test followed by the Tukey’s multiple comparisons test. Data were expressed as mean ± standard deviation (*P ≤ 0.05, **P ≤ 0.01, ***P ≤ 0.001, ****P ≤ 0.0001). The statistical differences represented by red horizontal bars and asterisks refer to the variation of neuronal density between TD groups treated with different doses of RSV. The gray horizontal bars and asterisks refer to the differences between CT and TD treated with the same doses of RSV. **D** Confocal microscopy images (×60) of OHC with markings of mature neurons (NeuN+) in red, cell nucleus (DAPI) in blue and activated microglia (Iba1+) in green (scale bar = 50 μm)

Figure 7 shows the activated microglia evidenced by Iba1 expression (Panel A) and typical cell morphology represented as endpoints/branch length ratio (Panel B), as well as the average density of neurons (Panel C) in OHCs at day 7 (day 3 of RSV treatment). RSV did not affect these two features in OHCs from the control group cultured with B1. However, RSV at 5 and 50 μM significantly increased the population of mature neurons and decreased microglia activation in B1 deprivation returning these two features to the control levels. These results confirm that neuroinflammation is indeed an inhibitory mechanism of hippocampal neurogenesis in TD.

## Discussion

OHCs were used to investigate the molecular mechanisms underlying the imbalance between neuronal death and neurogenesis associated with thiamine deficiency in the hippocampus. In OHCs cultured without B1, neuronal density decayed after 5 days and, at the 7th day, the epigenetic markings H3K4me3 and H3K9me3 were altered in mature neurons likely favoring gene transcription. Between the 7th and the 14th day of B1 deprivation, a pulse of neurogenesis was observed followed by a further massive neuron loss. Molecular analyses at later days of TD disclosed differentially expressed genes involved in inflammation reduction, metabolic shifting, maturation of new neurons and neurogenesis regulation. Finally, treating B1-deprived OHCs with RSV reduced microglia activation and induced and earlier pulse of neurogenesis. To the best of our knowledge, this is the first work to report a spontaneous and inflammation regulated hippocampal repopulation with new neurons in a TD model, opening a new search space for therapeutic approaches aiming at treating associated TD conditions.

### Unexpected neuron loss/neurogenesis dynamics in OHCs with TD

B1 is a cofactor of some enzymatic complexes that catalyze decarboxylation and transketolization reactions. The activity of the enzymatic complexes KGDHC and PDHC is impaired in the absence of B1, resulting in decreased ATP production, pyruvate accumulation, increased lactic acidosis and ROS production. These processes may culminate in cell membrane lesions, oxidative stress and activation of intracellular pathway signaling cascades linked to caspase-3-mediated apoptosis [4]. The decrease in KGDHC activity is further impaired by the production of nitric oxide (NO) and ROS [31]. Accordingly, the immunofluorescence analyses revealed that, between 4 and 7 days of B1 deprivation, there is a significant decrease in the neuronal population of the OHCs (Fig. 2A) and this massive neuron death is due to apoptosis (Fig. 1). While about 40% neuronal cell death was observed after 4 days of TD compared to controls for which there is almost no cell death, at this timepoint there is no difference in density of NeuN+-stained mature neurons between TD and control cultures. These results suggest that at day 4 of TD, apoptotic mature neurons still have nucleus capable of retaining the NeuN protein. Only from the 5th day on the decrease in this marking is detectable, probably as a result of the nuclear membrane disintegration in advanced apoptosis.

A pulse of neurogenesis between 7 and 10 days of thiamine deprivation was demonstrated combining BrdU incorporation assay and NeuN staining. This is the first study to report the spontaneous hippocampal repopulation with new neurons in a TD model. One hypothesis capable of explaining this phenomenon is that OHCs’ NPCs could survive B1 deprivation due to their quiescence [32], while mature neurons and glia would succumb prematurely due to their high energy expenditure. Nevertheless, once these stem cells form new neurons, they would probably become more vulnerable to the effects of TD, which could explain the subsequent massive decrease of neuron population 4 days after the neurogenesis pulse (Fig. 2A).

The concomitant increase of H3K4me3, associated with active transcription, and decrease of H3K9me3, associated with heterochromatin, in mature neurons from OHCs maintained for 7 days in B1 deprivation (Fig. 3) indicate a regulatory mechanism favoring gene expression. It is likely that this epigenetic reprogramming subsidizes the limitation of the neurodegenerative process induced by TD and also promotes the neurogenesis pulse observed between days 7 and 10 of B1 deprivation.

The transcriptional signature of OHCs cultured for 9 days in B1 deprivation includes the upregulated coding gene of the enzyme alpha-ketoglutarate dehydrogenase (OGDH) (Fig. 4 and Additional file 2) that catalyzes the conversion of alpha-ketoglutarate (αKG or 2OG) into succinyl-CoA and CO_2_ in the Krebs cycle. Previous studies indicate that the intermediates of the energy metabolism pathway can shape the epigenetic landscape of chromatin, regulating the methylation of CpGs and histones [33, 34]. In this scenario, αKG stands out for being a mandatory substrate for αKG-dependent dioxygenases. This family of enzymes includes the main demethylation proteins of CpGs and histones, i.e., TETs and JmjC (Jumonji C domain-containing) demethylases, respectively [35, 36]. Succinate and fumarate, also intermediates of the Krebs cycle, inhibits αKG-dependent dioxygenases, leading to increased methylation of CpGs and histones [37]. Thus, disturbances of Krebs cycle reactions, such as those caused by TD, can affect DNA and histone methylation. However, it is important to emphasize that the epigenetic regulation of gene expression is the result of numerous coexisting processes, such as other histone changes, and also methylation of CpGs. Thus, further analysis is needed to determine the epigenetic mechanisms related to the neuroregeneration phenotype reported herein.

### Reduced neuroinflammation and metabolic shift may regulate neurogenesis

#### Neuroinflammation reduction

Vemuganti et al. [38] demonstrated that inflammatory genes represent the largest functional group of positively regulated transcripts in regions affected by TD in the rat brain. In fact, oxidative stress due to energy collapse and excitotoxicity during TD leads to the activation of microglia and triggers the release of pro-inflammatory cytokines by regulating the signaling pathways of NFκB (Nuclear factor kappa B) and TNF-α [38–42]. In the present study, functional enrichment analysis of genes differentially expressed during the neurogenesis pulse in B1-deficient OHCs point out the canonical signaling pathways by TNF signaling and neuroinflammation signaling, in addition to the biological processes (GO) of response to TNF, inflammatory response and immune response as the most strongly inhibited (Additional files 3 and 4). The functional enrichment analysis also disclosed APP inhibition as the central regulator mechanism for neuroinflammation reduction in the late phase of TD in the OHCs model (Additional file 4). This is a membrane protein that, although expressed in many tissues, is especially abundant in the synapses of neurons. APP is a precursor of peptide Aβ, whose fibrillar form is the primary component of amyloid plaques, found in accumulation in the brain of patients affected by [43]. The accumulation of APP has already been reported as a response to TD, leading to the generation of Aβ by increasing the activity of β-secretases. This mechanism subsequently intensifies oxidative stress and neurodegeneration [44–46]. With the reduction of APP, inhibition of *Creb1*, *Nfkbia* (Nuclear factor-kappa-B-inhibitor alpha), *Stat3* (Signal transducer and activator of transcription 3), *Tlr2* (Toll-like receptor 2) and *Tlr4* (Toll-like receptor 4) (Additional file 4) continue the cascade of inflammation attenuation, inhibiting the expression of the genes “downstream” in OHCs after 9 days of culture without B1. In addition to APP, inhibition of NFE2L2, IFNG, TGFB1, CREB1 and TNF, seems to directly regulate the decrease in the inflammatory response of OHCs in TD (Additional file 4). Functional enrichment analysis also evidenced the activation of the PI3K–Akt signaling pathway in OHCs at the 9th day of TD. Class I PI3Ks induce NFκB activation and are involved in signal transduction by TLRs (Toll-like receptors) in immune cells, such as macrophages. However, different isoforms of PI3Ks class I may positively or negatively regulate the production of pro-inflammatory cytokines (reviewed in [47]). The remarkable downregulation of inflammatory genes observed in the period preceding the complete repopulation of OHCs with new neurons, in addition to the strong negative correlation between mRNA levels of inflammatory response gene *Mmp9* and neurogenesis and synaptogenesis genes *Neurod1* and *Bdnf* (Fig. 5 and Additional file 5) point towards neuroinflammation as a key negative regulator of neurogenesis during TD.

#### Metabolic shifting

The transcriptional signature of OHCs cultured for 9 days in B1 deprivation includes the upregulation of *Ogdh* and other genes whose products participate in pathways related to acetyl-CoA synthesis from amino acids (Additional file 2). This metabolic change could ultimately allow the OHCs to circumvent the energy bottleneck resulting from decreased activity of TK, KGDHC and PDHC due to the lack of B1, their cofactor. Through this mechanism, the Krebs cycle and the oxidative phosphorylation, which is the main source of energy of mature neurons [48] could be favored. However, immunoassays or enzyme activity studies are necessary to confirm this inference.

#### Maturation of new neurons

The maturation of the new neurons in OHCs maintained for 9 days in B1 deprivation seems to be regulated by the activation of the KEGG pathway PI3K/Akt signaling (Additional file 3). PI3Ks (phosphoinositide 3-kinases) are a family of enzymes that play a central role in several metabolic processes regulating various aspects of cellular physiology (reviewed in [49, 50]). PI3Ks are enzymes that transduce mitogenic and metabolic signals to promote cell growth, proliferation, migration and apoptosis. The PI3K class I family activates the protein kinase B (PKB), also known as AKT, which in turn activates mTOR (mammalian target of rapamycin) (reviewed in [49, 50]) In the hippocampus, cerebral cortex and cerebellum, activation of the AKT/mTOR pathway is essential for neuronal development and synapse formation [51–53] thus contributing to neuronal plasticity and memory [54–56]. The activation of this pathway by growth factors signaling through the tyrosine kinase receptor (RTK) constitutes the central regulatory path of neuronal proliferation, maturation and integration in mature circuits in the brain (reviewed in [57]).

PI3K, through the activation of AKT, increases the interaction and activity of several transcription complexes composed of transcription factors bHLH (basic helix-loop-helix) (NGN2 and NEUROD1), HATs and HDACs [58]. The activation of AKT by the natural flavonoid curcumin in combination with an HDACs inhibitor improves the survival of neurons in culture and restores neuronal damage induced by Aβ [59]. One of the mechanisms proposed for such protection is AKT-dependent phosphorylation of the CREB transcription factor, which in turn stimulates the expression of BDNF. A previous study suggested that PI3K/AKT is the signaling pathway responsible for the activation of late-phase gene expression involved in neuronal differentiation, neurogenesis and neuroprotection [60]. In addition, the NGF-dependent PI3K/AKT/NFκB signaling selectively regulates the trimethylation status of H3K9, favoring cell survival and neuronal differentiation [61].

The αKG-dependent enzymes, which are negatively regulated by the lack of substrate due to the imbalance of the Krebs cycle during TD, can also reduce collagen synthesis and promote hypoxia response [62]. Energy collapse and oxidative stress limit cell proliferation and reduce collagen synthesis. However, with the metabolic change inferred by the upregulation of genes related to the production of acetyl-CoA from amino acids on the 9th day of B1 deprivation, it appears that energy supply could be restored and oxidative stress could be brought under control. This could explain the collagen synthesis indicated by the increased expression of the genes *Col8a2* (Collagen type VIII alpha 2 chain), *Col11a2* (Collagen type XI alpha 2 chain) and *Colgalt2* (Collagen beta(1-*O*) galactosyltransferase 2) (Additional file 2). These genes have GO annotations related to cell adhesion, nervous system development and interaction between receptor and extracellular matrix. The activities of cell adhesion receptors, which mediate the interactions between the pre- and post-synaptic boutons, are largely influenced by the composition of the ECM. Cell adhesion is also essential to neuronal migration [63]. These results suggest a recovery of synaptic stability and plasticity in the OHCs at the 9th day of TD.

#### Neurogenesis regulation

The set of genes differentially expressed after 9 days of B1 deprivation points to a genetic programming in favor of the negative regulation of neurogenesis mediated by the trimethylation of H3 histone lysine 36. The higher expression of the *Nsd1* gene is related to the regulation of mono and demethylation of H3K36. The trimethylation of this residue, however, is catalyzed by the constitutive enzyme SET2 (SET domain-containing 2). The epigenetic marking H3K36me3 is related to the transcriptional activation of *Bmp4*, which in turn inhibits neurogenesis [64] (Fig. 6). The transcriptional profiling of OHCs also revealed inhibition of the Rassf10 (Ras association domain family member 10) (Additional file 2), which is involved in regulating cell proliferation and mitosis progression [65, 66]. Rassf10 downregulation in OHCs during TD may indicate a stimulus to cell survival. Finally, genes mapped in KEGG pathways related to the cell cycle, such as *Gadd45*, were found to be downregulated (Additional file 3). Reduced *Gadd45* expression is related to G2-M stop of the cell cycle and, in a way, to the suppression of cell growth [67]. Altogether, these results indicate that when OHCs under TD are completely repopulated with new neurons, cell proliferation is inhibited.

### Resveratrol promotes and earlier neurogenesis pulse in OHCs during TD

In order to test whether a causal relationship exists between the inflammatory response to TD and the inhibition of hippocampal neurogenesis, OHCs of the control and TD groups were treated with RSV starting at day 4 of culture, the day before the observed onset of neurodegeneration caused by TD in OHCs. RSV is an anti-inflammatory and antioxidant polyphenol found mainly in the seeds and films of grapes and red wine, red fruits and peanuts (reviewed in [68]). In a dose dependent manner, RSV at 5 and 50 µM, but not at 100 µM, prevented microglia activation and promoted and earlier neurogenesis pulse in B1-deficient OHCs, adding confidence to the hypothesis that decreasing neuroinflammation is the trigger to the neurogenesis pulse in the OHC model of TD (Fig. 7). There are evidence that RSV decreases the amyloidogenic cleavage of APP, enhances Aβ clearance and reduces its aggregation [69]. However, it must be taken into account that, in addition to its anti-inflammatory and antioxidant properties, RSV also upregulates *Bdnf* transcription in the hippocampus [70–75].

Previous studies have reported neuroprotective effects of other anti-inflammatory drugs in animal models of TD. For instance, adjuvant therapy with the non-steroidal anti-inflammatory dimethyl sulfoxide (DMSO) associated with B1 replacement attenuated neurological disorders and cellular disfunction in a murine model of TD [76]. Furthermore, minocycline, an antibiotic with anti-inflammatory properties prevented the activation of microglia and delayed the onset of neurological alterations, such as the loss of the righting reflex, by approximately 39 h in rats with TD [39]. However, this is the first study to demonstrate the association between inflammation and the control of hippocampal neurogenesis in TD, opening a new search space for therapeutic approaches aiming at promoting neuroregeneration in WKS.

The use of the thiamine antagonist pyrithiamine to induce TD is generally considered to be a more realistic modeling of WKS in rodents than simple thiamine deprivation. However, thiamine antagonists were not used in this study because they directly trigger apoptosis in neurons [77] and could mask the effect of TD reported herein by simply culturing the OHCs without the vitamin. A limitation of this OHC model is that over time the typical organ architecture is lost in the OHCs and hippocampal regions are no longer recognizable.

## Conclusion

**Fig. 8:**
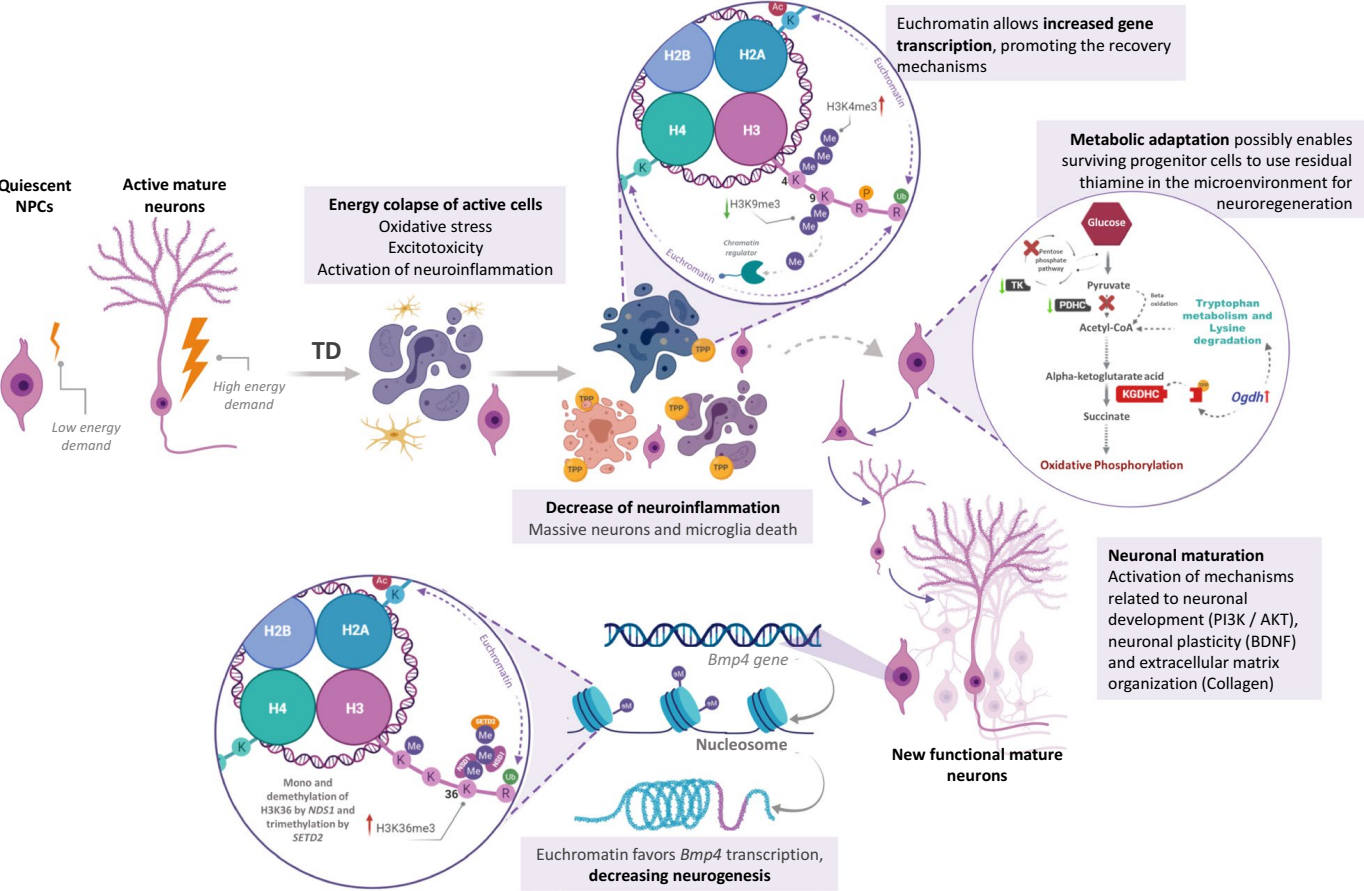
Neuroinflammation regulate the balance between hippocampal neuron death and neurogenesis in TD. NPC: neural progenitor cells; TD: thiamine deficiency; TPP thiamine pyrophosphate; H2A: histone 2A; H2B: histone 2B; H3: histone 3; H4: histone 4; K: lysine; R: arginine; P: phosphorylation; Ub: ubiquitination; Ac: acetylation; Me: methylation; H3K9me3: trimethylation in lysine 9 at histone 3; K3K4me3: trimethylation in lysine 4 at histone 3; TK: transketolase; PDHC: pyruvate dehydrogenase complex; *Ogdh*: oxoglutarate dehydrogenase; KGDHC: alfa-ketoglutarate dehydrogenase complex; PI3K/AKT: phosphatidylinositol 3-kinase/protein kinase B; BDNF: brain derived neurotrophic factor; H3K36me3: trimethylation in lysine 36 at histone 3; NSD1: nuclear receptor binding SET domain protein 1; SETD2: SET domain containing 2; *Bmp4*: bone morphogenetic protein 4

The main contribution of this work is the unprecedented report of a spontaneous neurogenesis pulse in the hippocampus during an advanced stage of B1 deficiency (Fig. 8). This phenomenon is triggered by the reduction of neuroinflammation. The high energy demand for hippocampal repopulation with new neurons depends on the metabolic shifting towards oxidative phosphorylation, which is likely to be mediated by the upregulation of genes from pathways involved in the production of acetyl-CoA from amino acids to circumventing the bottleneck in the Krebs cycle caused by the lack of B1. Chromatin remodeling by histone methylation seems to play a role in the regulation of these processes. Thus, neuroinflammation is implicated as a major regulator of hippocampal neurogenesis in TD opening a new search space for therapeutic approaches aiming at treating WKS.

## Supplementary Information

**Additional file 1.** Central energy metabolism. ATP: adenosine triphosphate; NAD+: nicotinamide adenine dinucleotide (oxidized); NADH: nicotinamide adenine dinucleotide (reduced); NADPH: nicotinamide adenine dinucleotide phosphate; H^+^: hydrogen (proton); CoA: coenzyme A; TPP: thiamine pyrophosphate; Pi: inorganic phosphate group; CO_2_: carbon dioxide, H_2_O: water, FAD: flavin-adenine dinucleotide; FADH_2_: flavin-adenine dinucleotide (hydroquinone form). Created with BioRender.com.

**Additional file 2.** Differentially expressed genes in OHCs cultured for 9 days in TD compared to the controls. List of genes with increased or reduced expression in OHCs in response to 9 days of TD, found in contrast analysis with DESeq2 software. Genes with Fold Change greater than 1.5 and adjusted *P* value lower than 0.01 were considered as differentially expressed. UNIPROT identifiers, protein names and gene symbols were obtained with DAVID web-software using Ensembl identifiers.

**Additional file 3.** Functional enrichment analysis of the 89 differentially expressed genes using the Database for Annotation, Visualization and Integrated Discovery (DAVID) software. Considered only molecules and/or relationships where (species = *Rattus norvegicus*). Predictions with *P* value lower than 0.05 in Fisher’s test were considered statistically significant.

**Additional file 4.** Functional enrichment analysis of the 89 differentially expressed genes using the Ingenuity Pathway Analysis (IPA) software. Considered only molecules and/or relationships where (species = Rat) AND (confidence = experimentally observed or highly predicted) AND (tissues/ cell lineage = Hippocampus OR all CNS cell lines OR UNSPECIFIED CNS lines). Predictions with *P* value lower than 0.05 in Fisher’s test were considered statistically significant.

**Additional file 5.** Pearson’s correlation coefficients between the expression level (mRNA) of selected genes altered by TD after 4, 7 and 9 days in OHCs. Correlations with *P* value lower than 0.05 were considered statistically significant.

## Data Availability

The dataset supporting the conclusions of this article is available in the SRA repository, (http://www.ncbi.nlm.nih.gov/bioproject/806989).

## Abbreviations

ATP: Adenosine triphosphate
Aβ: Amyloid beta
B1: Vitamin B1 (thiamine)
CNS: Central nervous system
CpG: Cytosine nucleotide followed by a guanine
DEG: Differentially expressed gene
ECM: Extracellular matrix
GO: Gene Ontology
IPA: Ingenuity Pathways Analysis
KEGG: Kyoto Encyclopedia of Genes and Genomes
KGDHC: α-Ketoglutarate dehydrogenase complex
NO: Nitric oxide
NPC: Neural progenitor cells
OHC: Organotypic hippocampal slice culture
PDHC: Pyruvate dehydrogenase complex
ROS: Reactive oxygen species
RSV: Resveratrol
TD: Thiamine deficiency
TK: Transketolase
TPP: Thiamine pyrophosphate
WE: Wernicke’s encephalopathy
WKS: Wernicke–Korsakoff syndrome
