# Supplementary material for: Neuroinflammation regulates the balance between hippocampal neuron death and neurogenesis in an ex vivo model of thiamine deficiency
Source: J Neuroinflammation. 2022 Nov 14;19:272. doi: 10.1186/s12974-022-02624-6 (PMC9664832; doi:10.1186/s12974-022-02624-6)

**Additional File 1 – Central energy metabolism**

ATP = Adenosine triphosphate, NAD+ = Nicotinamide adenine dinucleotide (oxidized), NADH = Nicotinamide adenine dinucleotide (reduced), NADPH = Nicotinamide adenine dinucleotide phosphate, H+ = Hydrogen (proton), CoA = Coenzyme A, TPP = Thiamine pyrophosphate, Pi = Inorganic phosphate group, CO_2_ = Carbon dioxide, H_2_O, = Water, FAD = Flavin-adenine dinucleotide, FADH_2_ = Flavin-adenine dinucleotide (hydroquinone form). Created with BioRender.com.


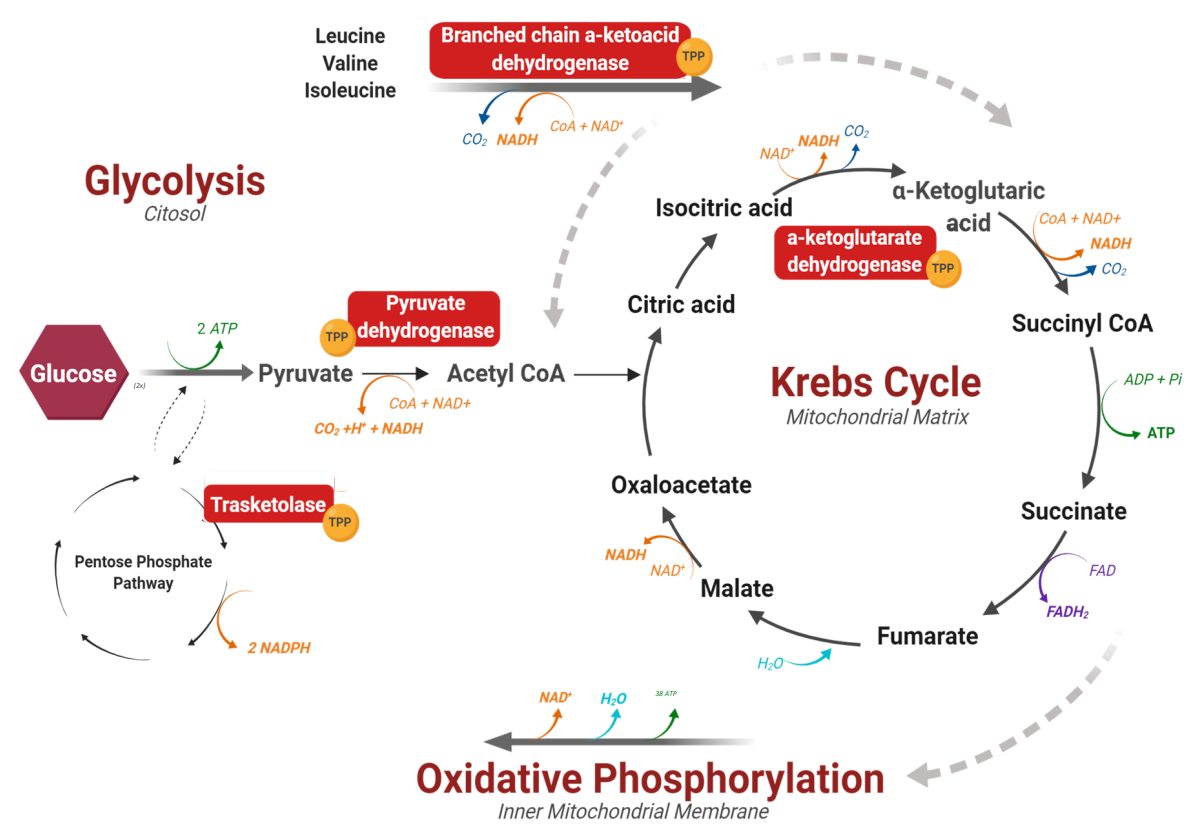

Supplement: Supplementary file 1 — Additional file 1. Central energy metabolism. ATP: adenosine triphosphate; NAD+: nicotinamide adenine dinucleotide (oxidized); NADH: nicotinamide adenine dinucleotide (reduced); NADPH: nicotinamide adenine dinucleotide phosphate; H+: hydrogen (proton); CoA: coenzyme A; TPP: thiamine pyrophosphate; Pi: inorganic phosphate group; CO2: carbon dioxide, H2O: water, FAD: flavin-adenine dinucleotide; FADH2: flavin-adenine dinucleotide (hydroquinone form). Created with BioRender.com. [file 12974_2022_2624_MOESM1_ESM.docx]
