# Supplementary material for: Neuroinflammation regulates the balance between hippocampal neuron death and neurogenesis in an ex vivo model of thiamine deficiency
Source: J Neuroinflammation. 2022 Nov 14;19:272. doi: 10.1186/s12974-022-02624-6 (PMC9664832; doi:10.1186/s12974-022-02624-6)
Supplement: Supplementary file 2 — Additional file 2. Differentially expressed genes in OHCs cultured for 9 days in TD compared to the controls. List of genes with increased or reduced expression in OHCs in response to 9 days of TD, found in contrast analysis with DESeq2 software. Genes with Fold Change greater than 1.5 and adjusted P value lower than 0.01 were considered as differentially expressed. UNIPROT identifiers, protein names and gene symbols were obtained with DAVID web-software using Ensembl identifiers. [file 12974_2022_2624_MOESM2_ESM.docx]

Additional File 2 - Differentially expressed genes in OHCs cultured for nine days in TD, compared to the controls

| **Ensembl ID** | ***Gene symbol*** | **Protein name** | **UNIPROT ID** | **Fold Change** | **Adj *P value*** |
| --- | --- | --- | --- | --- | --- |
| ENSRNOG00000000463 | *Col11a2* | Collagen type XI alpha 2 chain | F6T0B3 | 1.51025 | 3.04E-03 |
| ENSRNOG00000001469 | *Eln* | Elastin | D4A9U4 | 1.53873 | 3.21E-03 |
| ENSRNOG00000002050 | *Igfbp7* | Insulin-like growth factor binding protein 7 | F1M9B2 | 0.57916 | 4.45E-08 |
| ENSRNOG00000002468 | *Tnr* | Tenascin-R | F1LQ63 | 1.57160 | 1.74E-03 |
| ENSRNOG00000002843 | *Cxcl6* | C-X-C motif chemokine 6 | G3V6C8 | 0.57205 | 1.18E-05 |
| ENSRNOG00000003537 | *Spta1* | Spectrin | D4A678 | 2.35701 | 2.56E-35 |
| ENSRNOG00000003887 | *Lgi2* | Leucine-rich repeat LGI family member 2 | F7ESR8 | 0.48128 | 4.17E-10 |
| ENSRNOG00000004861 | *Itga4* | Integrin subunit alpha 4 | D3ZMQ3 | 1.61018 | 7.89E-04 |
| ENSRNOG00000005130 | *Ogdh* | 2-oxoglutarate dehydrogenase complex component E1 | Q5XI78 | 1.54902 | 1.80E-08 |
| ENSRNOG00000005731 | *Birc3* | Baculoviral IAP repeat-containing 3 | F7FLN8 | 0.65789 | 4.33E-03 |
| ENSRNOG00000005830 | *Fam19a4* | Fam19a4 protein | B1H244 | 0.64612 | 1.59E-04 |
| ENSRNOG00000006735 | *Cdkn2b* | Cyclin-dependent kinase 4 inhibitor B | Q5PQW4 | 0.64041 | 2.07E-03 |
| ENSRNOG00000006956 | *AABR07049085.1* | Uncharacterized protein | F1M3T9 | 0.62867 | 1.16E-03 |
| ENSRNOG00000007319 | *Trib3* | Tribbles homolog 3 | Q9WTQ6 | 0.63385 | 1.24E-03 |
| ENSRNOG00000007457 | *Serping1* | Plasma protease C1 inhibitor | Q6P734 | 0.56281 | 2.48E-09 |
| ENSRNOG00000008001 | *Rab3b* | Ras-related protein Rab-3B | Q63941 | 0.61693 | 8.02E-05 |
| ENSRNOG00000008173 | *Sesn3* | Sestrin 3 | D4A469 | 1.51102 | 4.89E-03 |
| ENSRNOG00000008178 | *Nxn* | Nucleoredoxin | D4A0M2 | 0.65544 | 1.93E-09 |
| ENSRNOG00000008257 | *Mfap2* | Microfibril-associated protein 2 | D3Z952 | 1.70876 | 1.62E-05 |
| ENSRNOG00000008602 | *Steap4* | Metalloreductase 4 | Q4V8K1 | 0.49561 | 2.03E-09 |
| ENSRNOG00000008615 | *Mal2* | Mal T-cell differentiation protein 2 | Q7TPB7 | 0.65474 | 4.68E-03 |
| ENSRNOG00000008622 | *Creb5* | cAMP-responsive element-binding protein 5 | D3ZBH0 | 1.55331 | 3.55E-04 |
| ENSRNOG00000008798 | *Pipox* | Pipecolic acid oxidase | Q5I0K1 | 0.64151 | 2.16E-03 |
| ENSRNOG00000009253 | *Igsf9b* | Immunoglobulin superfamily member 9B | D3ZB51 | 1.57963 | 1.43E-03 |
| ENSRNOG00000009620 | *Cybrd1* | Cytochrome b reductase 1 | Q5RKJ2 | 2.08759 | 2.34E-22 |
| ENSRNOG00000009980 | *Plpp1* | Phospholipid phosphatase 1 | G3V9Y2 | 0.65704 | 7.87E-09 |
| ENSRNOG00000010047 | *Ddit4l* | DNA damage-inducible transcript 4-like protein | Q8VD50 | 1.79506 | 8.18E-11 |
| ENSRNOG00000010454 | *Ccno* | Cyclin O | D3ZHT5 | 0.61105 | 3.55E-04 |
| ENSRNOG00000010478 | *Serpina3n* | Serine protease inhibitor A3N | A0A0H2UHI5 | 0.35853 | 3.28E-24 |
| ENSRNOG00000010841 | *Col8a2* | Collagen type VIII alpha 2 chain | D4ADG9 | 1.62377 | 5.33E-04 |
| ENSRNOG00000012031 | *St8sia2* | Alpha-2,8-sialyltransferase 8B | Q07977 | 1.69166 | 3.63E-05 |
| ENSRNOG00000012235 | *Ppp1r17* | Protein phosphatase 1, regulatory subunit 17 | Q8CJC8 | 0.57387 | 3.51E-07 |
| ENSRNOG00000012280 | *Ptx3* | Pentraxin 3 | D3ZT94 | 0.65833 | 8.63E-04 |
| ENSRNOG00000012302 | *Gucy1a1* | Guanylate cyclase soluble subunit alpha-1 | Q5U330 | 0.63848 | 2.07E-03 |
| ENSRNOG00000012772 | *Nqo1* | NAD(P)H dehydrogenase [quinone] 1 | P05982 | 1.51861 | 3.18E-03 |
| ENSRNOG00000013484 | *Gsta3* | Glutathione S-transferase alpha-3 | P04904 | 1.52838 | 1.74E-03 |
| ENSRNOG00000013729 | *RGD1306271* | Similar to KIAA1549 protein | D3Z9D0 | 1.55242 | 4.72E-05 |
| ENSRNOG00000013820 | *Tnfrsf14* | Tumor necrosis factor receptor superfamily, member 14 | Q5BK53 | 0.64822 | 2.07E-03 |
| ENSRNOG00000013917 | *Igsf10* | Immunoglobulin superfamily member 10 | G3V7S1 | 1.64161 | 4.05E-05 |
| ENSRNOG00000013973 | *Lcn2* | Lipocalin-2 | P30152 | 0.35529 | 5.19E-22 |
| ENSRNOG00000014046 | *Sertm1* | Serine-rich and transmembrane domain-containing 1 | D3ZR22 | 0.61655 | 3.88E-04 |
| ENSRNOG00000014314 | *Slc39a4* | Solute carrier family 39 member 4 | A0A0H2UHY4 | 1.69177 | 8.46E-05 |
| ENSRNOG00000014371 | *Cdh13* | Cadherin 13 | F1M7X3 | 0.45024 | 4.44E-14 |
| ENSRNOG00000014847 | *Rassf10* | Ras association domain family member 10) | A0A0G2JT12 | 0.61110 | 4.19E-04 |
| ENSRNOG00000014948 | *Osgin1* | Oxidative stress-induced growth inhibitor | Q8R430 | 1.50533 | 1.88E-03 |
| ENSRNOG00000015055 | *Scg2* | Secretogranin-2 | G3V7X2 | 0.64374 | 2.02E-03 |
| ENSRNOG00000015354 | *Aox1* | Aldehyde oxidase 1 | F1LRQ1 | 1.61296 | 1.03E-04 |
| ENSRNOG00000016581 | *Serpinb1a* | Serine protease inhibitor family B member 1A | Q4G075 | 0.66141 | 4.65E-03 |
| ENSRNOG00000016680 | *Nsd1* | Nuclear receptor binding SET domain protein 1 | D4AA06 | 1.62707 | 3.90E-05 |
| ENSRNOG00000016945 | *Pla2g2a* | Phospholipase A2 group IIA | P14423 | 0.55398 | 1.66E-07 |
| ENSRNOG00000017206 | *Igfbp5* | Insulin-like growth factor-binding protein 5 | P24594 | 2.04585 | 1.66E-12 |
| ENSRNOG00000017409 | *Wnt6* | Protein Wnt | D4A3W9 | 0.65202 | 4.30E-03 |
| ENSRNOG00000017539 | *Mmp9* | Matrix Metalloproteinase 9 | D3ZYK8 | 0.54275 | 6.51E-07 |
| ENSRNOG00000019048 | *Sod2* | Superoxide dismutase [Mn], mitochondrial | P07895 | 0.62230 | 2.28E-04 |
| ENSRNOG00000019120 | *Hmgcs2* | 3-hydroxy-3-methylglutaryl coenzyme A synthase | Q68G44 | 0.57495 | 4.45E-08 |
| ENSRNOG00000019822 | *Gadd45b* | Growth arrest and DNA-damage-inducible 45 beta | Q5U3Z2 | 0.66128 | 5.61E-04 |
| ENSRNOG00000019500 | *Cyp1a1* | Cytochrome P450 1A1 | P00185 | 1.55644 | 2.37E-03 |
| ENSRNOG00000020277 | *Cntnap1* | Contactin-associated protein 1 | P97846 | 1.55296 | 1.69E-04 |
| ENSRNOG00000020482 | *Nfatc4* | Nuclear factor of-activated T-cells 4 | A0A0G2K0L1 | 1.59888 | 8.80E-04 |
| ENSRNOG00000021105 | *Gabpb2* | GA-binding protein transcription factor subunit beta 2 | D3ZSD3 | 1.50081 | 1.43E-03 |
| ENSRNOG00000022681 | *RGD1561113* | Similar to Hypothetical UPF0184 protein | D3ZBT2 | 0.65584 | 1.67E-03 |
| ENSRNOG00000022919 | *Chst8* | Carbohydrate sulfotransferase | B1WBV7 | 0.59046 | 4.27E-05 |
| ENSRNOG00000023548 | *Sned1* | Sushi, nidogen and EGF-like domains 1 | D3ZNR4 | 1.52444 | 4.65E-03 |
| ENSRNOG00000023760 | *Plekhm3* | Pleckstrin homology domain-containing M3 | D4A959 | 1.60265 | 1.58E-05 |
| ENSRNOG00000024899 | *Cxcl13* | C-X-C motif chemokine ligand 13 | F7F7W7 | 0.56468 | 1.47E-12 |
| ENSRNOG00000025589 | *Jph4* | Junctophilin-4 | A0A0A0MY08 | 1.64601 | 3.13E-04 |
| ENSRNOG00000026110 | *Scml4* | Scm polycomb group protein-like 4 | A0A0G2K2K4 | 1.51179 | 6.23E-03 |
| ENSRNOG00000028207 | *Colgalt2* | Collagen beta(1-O) galactosyltransferase 2 | D3Z9Z7 | 1.58478 | 3.55E-04 |
| ENSRNOG00000029768 | *Ccl12* | C-C motif chemokine 12 | D4ABS1 | 0.62095 | 7.74E-04 |
| ENSRNOG00000031163 | *Nfkbiz* | NFKB inhibitor zeta | A0A0G2K6U7 | 0.66318 | 1.18E-03 |
| ENSRNOG00000031167 | *AABR07054319.1* | Sulfiredoxin 1 | Q7TP44 | 1.85022 | 4.17E-10 |
| ENSRNOG00000032002 | *Hapln1* | Hyaluronan and proteoglycan link protein 1 | P03994 | 1.60636 | 2.40E-04 |
| ENSRNOG00000037621 | *Spata48* | Spermatogenesis associated 48 | D4A9H9 | 1.68897 | 1.20E-04 |
| ENSRNOG00000045989 | *Hba-a3* | Hemoglobin alpha, adult chain 3 | Q63910 | 0.63567 | 1.34E-04 |
| ENSRNOG00000046699 | *Slpi* | Secretory leukocyte peptidase inhibitor | A0A096MJ68 | 0.64224 | 8.70E-04 |
| ENSRNOG00000046834 | *C3* | Complement C3 | M0RBJ7 | 0.55144 | 3.01E-06 |
| ENSRNOG00000047450 | *Lmo3* | LIM domain only protein 3 | M0R4L0 | 1.59832 | 9.80E-04 |
| ENSRNOG00000048088 | *Mest* | Mesoderm-specific transcript homolog protein | M0R830 | 1.59825 | 2.24E-05 |
| ENSRNOG00000050206 | *Shank2* | SH3 and multiple ankyrin repeat domains protein 2 | Q9QX74 | 1.89099 | 3.51E-07 |
| ENSRNOG00000052129 | *Nwd1* | NACHT and WD repeat domain containing 1 | A0A0G2JWP6 | 1.55298 | 6.80E-05 |
| ENSRNOG00000053272 | *Chi3l1* | Chitinase-3-like protein 1 | Q9WTV1 | 0.56284 | 5.63E-07 |
| ENSRNOG00000054314 | *Kcng1* | Potassium voltage-gated channel subfamily G member 1 | D4AD53 | 1.56790 | 1.98E-03 |
| ENSRNOG00000054458 | *Kcnmb4* | Calcium-activated potassium channel subunit beta-4 | Q9ESK8 | 0.64448 | 1.18E-03 |
| ENSRNOG00000055078 | *Cyp4b1* | Cytochrome P450 4B1 | P15129 | 0.52021 | 1.26E-07 |
| ENSRNOG00000056457 | *Gpd1* | Glycerol-3-phosphate dehydrogenase | O35077 | 0.55874 | 8.45E-08 |
| ENSRNOG00000057221 | *Scn3b* | Sodium channel subunit beta-3 | Q9JK00 | 1.50474 | 1.55E-04 |
| ENSRNOG00000059837 | *Cdkn2a* | Tumor suppressor ARF | A0A0G2K211 | 0.60227 | 2.28E-04 |
| ENSRNOG00000061215 | *Crym* | Ketimine reductase mu-crystallin | A0A0G2K568 | 0.52656 | 4.36E-08 |
| ENSRNOG00000062276 | *AABR07044631.2* | Uncharacterized protein (Fragment) | A0A1W2Q6I6 | 1.68223 | 1.18E-05 |

List of genes with increased or reduced expression in OHCs in response to nine days of TD, found in contrast analysis with DESeq2 software. Genes with Fold Change greater than 1.5 and adjusted *P* value less than 0.01 were considered as differentially expressed. UNIPROT identifiers, protein names and gene symbols were obtained with the DAVID web-software using Ensembl identifiers.
