# Supplementary material for: Neuroinflammation regulates the balance between hippocampal neuron death and neurogenesis in an ex vivo model of thiamine deficiency
Source: J Neuroinflammation. 2022 Nov 14;19:272. doi: 10.1186/s12974-022-02624-6 (PMC9664832; doi:10.1186/s12974-022-02624-6)
Supplement: Supplementary file 3 — Additional file 3. Functional enrichment analysis of the 89 differentially expressed genes using the Database for Annotation, Visualization and Integrated Discovery (DAVID) software. Considered only molecules and/or relationships where (species = Rattus norvegicus). Predictions with P value lower than 0.05 in Fisher’s test were considered statistically significant. [file 12974_2022_2624_MOESM3_ESM.docx]

**Additional File 3 - Functional enrichment analysis of the 89 differentially expressed genes using the Database for Annotation, Visualization and Integrated Discovery (DAVID) software**

Parameterization:

Considered only molecules and/or relationships where (species = *Rattus norvegicus*)

Predictions with *P* value lower than 0.05 in Fisher’s test were considered statistically significant.

Enrichment analysis for canonical pathways:

| **KEGG Pathways** | **Mapped genes** | ***P value*** |
| --- | --- | --- |
| Tryptophan metabolism | *Aox1, Cyp1a1, Ogdh* | 3.00E-03 |
| Lysine Degradation | *Ogdh, Nsd1, Colgalt2* | 5.00E-03 |
| Cell cycle | *Ink4a, Ink4b, Arf, Gadd45* | 5.00E-02 |
| ECM-receptor interaction | *Col11a2, Tnr* | 1.00E-02 |
| PI3K-Akt signaling | *Creb, Ecm, Itga, Itgb* | 3.00E-02 |
| TNF Signaling | *Ccl12, Mmp9, Birc3* | 4.00E-02 |

List of the pathways whose components are with increased expression (red) or reduced (blue) in OHCs maintained in TD for nine days compared to the control.

Enrichment analysis of GO annotations for Biological Process:

| **Biological Process** | **Mapped genes** | ***P value*** |
| --- | --- | --- |
| Inflammatory response | *Cxcl13, Cxcl6, Nfkbia, Tnfrsf14, Ccl12, Chi3l1, C3, Serpina3n* | 6.00E-06 |
| Cellular response to TNF | *Birc3, Ccl12, Chi3l1, Gpd1, Lcn2 e Mmp9* | 1.00E-05 |
| Cellular response to IL-1 | *Ccl12, Chi3l1, Lcn2, Mmp9, Serpina3n* | 8.00E-05 |
| Chemokine-mediated signaling pathway | *Cxcl13, Cxcl6 e Ccl12* | 8.00E-03 |
| Immune response | *Cxcl13, Cxcl6, Tnfrsf14, Slpi* | 2.00E-02 |
| Nervous system development | *Igsf9b, Scn3b, Tnr* | 5.00E-02 |
| Cell adhesion | *Cntnap1, Hapln1, Itga4, Tnr* | 1.00E-02 |

List of GO annotations related to genes with increased expression (red) or reduced (blue) in OHCs maintained in TD for nine days compared to the control.
