# Supplementary material for: Neuroinflammation regulates the balance between hippocampal neuron death and neurogenesis in an ex vivo model of thiamine deficiency
Source: J Neuroinflammation. 2022 Nov 14;19:272. doi: 10.1186/s12974-022-02624-6 (PMC9664832; doi:10.1186/s12974-022-02624-6)
Supplement: Supplementary file 4 — Additional file 4. Functional enrichment analysis of the 89 differentially expressed genes using the Ingenuity Pathway Analysis (IPA) software. Considered only molecules and/or relationships where (species = Rat) AND (confidence = experimentally observed or highly predicted) AND (tissues/ cell lineage = Hippocampus OR all CNS cell lines OR UNSPECIFIED CNS lines). Predictions with P value lower than 0.05 in Fisher’s test were considered statistically significant. [file 12974_2022_2624_MOESM4_ESM.docx]

Additional File 4 - Functional enrichment analysis of the 89 differentially expressed genes using the Ingenuity Pathway Analysis (IPA) software.

Parameterization:

Considered only molecules and/or relationships where (species = Rat) AND (confidence = experimentally observed or highly predicted) AND (tissues/ cell lineage = Hippocampus OR all CNS cell lines OR UNSPECIFIED CNS lines)

Predictions with *P* value lower than 0.05 in Fisher’s test were considered statistically significant.

Enrichment analysis for canonical pathways:

| **Canonical Pathways** | **Mapped Genes** | ***P value*** |
| --- | --- | --- |
| Neuroinflammation Signaling | *Birc3, Ccl2, Creb5, Mmp9, Nfatc4, Sod2* | 1.00E-03 |
| 2-ketoglutarate dehydrogenase complex | *Ogdh* | 2.00E-02 |

List of the pathways whose components are with increased expression (red) or reduced (blue) in OHCs maintained in TD for nine days compared to the control.

Prediction of upstream regulators:

| **Regulators** | **Predicted as** | **Target genes** | ***P value*** |
| --- | --- | --- | --- |
| NFE2L2 | Inhibited | *Cxcl6, Kcnb4, Scg2, Nqo1* | 1.88E-04 |
| CREB1 | Inhibited | *Lcn2, Gadd45b, Lmo3, Igsf9b, Crym, Scg2* | 2.00E-05 |
| IFNG | Inhibited | *Ccl12, Chst8, Eln, Lcn2, Ptx3, Sod2* | 4.70E-04 |
| TNF | Inhibited | *Ccl12, Itga4, Lcn2, Mmp9, Sod2* | 2.00E-05 |
| TGFB1 | Inhibited | *Ccl12, Chst8, Eln, Itga4, Lcn2, Ptx3* | 5.00E-05 |
| APP | Inhibited | *Cxcl13, C3, Ccl12, Crym, Eln, Mmp9, Plpp1* | 1.45E-02 |

List of proteins predicted as target gene regulators with increased expression (red) or reduced (blue) in OHCs maintained in TD for nine days compared to control.

Prediction of causal regulators:

| **Central regulator** | **Downstream**  **regulators** | **Target genes** | ***P value*** |
| --- | --- | --- | --- |
| APP | *Creb1, Nfkbia, Stat3, Tlr2, Tlr4* | *Ccl12, C3, Chi3l1, Eln, Igsf9b, Gadd45b, Lcn2, Lmo3, Mmp9, Plpp1, Ptx3, Scg2, Sod2, Srxn1* | 5.33E-09 |

List of regulators predicted as inhibited (green) causally related to the alteration of gene expression whose expression was increased (red) or reduced (blue) in OHCs maintained in TD for nine days compared to control.
