# Supplementary material for: Neuroinflammation regulates the balance between hippocampal neuron death and neurogenesis in an ex vivo model of thiamine deficiency
Source: J Neuroinflammation. 2022 Nov 14;19:272. doi: 10.1186/s12974-022-02624-6 (PMC9664832; doi:10.1186/s12974-022-02624-6)
Supplement: Supplementary file 5 — Additional file 5. Pearson’s correlation coefficients between the expression level (mRNA) of selected genes altered by TD after 4, 7 and 9 days in OHCs. Correlations with P value lower than 0.05 were considered statistically significant. [file 12974_2022_2624_MOESM5_ESM.docx]

Additional File 5 – Pearson’s correlation coefficients between the expression levels (mRNA) of selected genes altered by TD after four, seven and nine days in OHCs.

| \|  \| ***Mmp9*** \| ***C3*** \| ***Lcn2*** \| ***Ogdh*** \| ***NeuroD1*** \| ***Bdnf*** \| ***Nsd1*** \| ***Bmp4*** \| \| --- \| --- \| --- \| --- \| --- \| --- \| --- \| --- \| --- \| \| ***Mmp9*** \| 1 \| 0.570 \| 0.537 \| -0.998* \| -0.997* \| -0.999** \| -0.985 \| -0.996* \| \| ***C3*** \|  \| 1 \| 0.999* \| -0.517 \| -0.507 \| -0.583 \| -0.418 \| -0.499 \| \| ***Lcn2*** \|  \|  \| 1 \| -0.483 \| -0.472 \| -0.551 \| -0.382 \| -0.465 \| \| ***Ogdh*** \|  \|  \|  \| 1 \| 0.999** \| 0.997* \| 0.994 \| 0.999* \| \| ***NeuroD1*** \|  \|  \|  \|  \| 1 \| 0.996* \| 0.995 \| 1.000** \| \| ***Bdnf*** \|  \|  \|  \|  \|  \| 1 \| 0.982 \| 0.995 \| \| ***Nsd1*** \|  \|  \|  \|  \|  \|  \| 1 \| 0.996* \| \| ***Bmp4*** \|  \|  \|  \|  \|  \|  \|  \| 1 \| |  |  |  |  |  |  |  |  |  |
| --- | --- | --- | --- | --- | --- | --- | --- | --- | --- | --- | --- | --- | --- | --- | --- | --- | --- | --- | --- | --- | --- | --- | --- | --- | --- | --- | --- | --- | --- | --- | --- | --- | --- | --- | --- | --- | --- | --- | --- | --- | --- | --- | --- | --- | --- | --- | --- | --- | --- | --- | --- | --- | --- | --- | --- | --- | --- | --- | --- | --- | --- | --- | --- | --- | --- | --- | --- | --- | --- | --- | --- | --- | --- | --- | --- | --- | --- | --- | --- | --- | --- | --- | --- | --- | --- | --- | --- | --- | --- | --- |
| (* = *P* ≤ 0.05, ** = *P* ≤ 0.01) |  |  |  |  |  |  |  |  |  |
